# Role of O-linked N-acetylglucosamine protein modification in oxidative stress-induced autophagy: a novel target for bone remodeling

**DOI:** 10.1186/s12964-024-01734-3

**Published:** 2024-07-10

**Authors:** Shengqian Li, Wenhao Ren, Jingjing Zheng, Shaoming Li, Keqian Zhi, Ling Gao

**Affiliations:** 1 Department of Oral and Maxillofacial Reconstruction, the Affiliated Hospital of Qingdao University, Qingdao, 266555 China; 2 School of Stomatology, Qingdao University, Qingdao, 266003 China; 3 Department of Endodontics, the Affiliated Hospital of Qingdao University, Qingdao, 266003 China; 4 Key Laboratory of Oral Clinical Medicine, the Affiliated Hospital of Qingdao University, Qingdao, 266003 China; 5 Department of Oral and Maxillofacial Surgery, the Affiliated Hospital of Qingdao University, Qingdao, 266555 China

**Keywords:** O-GlcNAcylation, Autophagy, Oxidative stress, Bone remodeling

## Abstract

O-linked N-acetylglucosamine protein modification (O-GlcNAcylation) is a dynamic post-translational modification (PTM) involving the covalent binding of serine and/or threonine residues, which regulates bone cell homeostasis. Reactive oxygen species (ROS) are increased due to oxidative stress in various pathological contexts related to bone remodeling, such as osteoporosis, arthritis, and bone fracture. Autophagy serves as a scavenger for ROS within bone marrow-derived mesenchymal stem cells, osteoclasts, and osteoblasts. However, oxidative stress-induced autophagy is affected by the metabolic status, leading to unfavorable clinical outcomes. O-GlcNAcylation can regulate the autophagy process both directly and indirectly through oxidative stress-related signaling pathways, ultimately improving bone remodeling. The present interventions for the bone remodeling process often focus on promoting osteogenesis or inhibiting osteoclast absorption, ignoring the effect of PTM on the overall process of bone remodeling. This review explores how O-GlcNAcylation synergizes with autophagy to exert multiple regulatory effects on bone remodeling under oxidative stress stimulation, indicating the application of O-GlcNAcylation as a new molecular target in the field of bone remodeling.

**Graphical Abstract:**

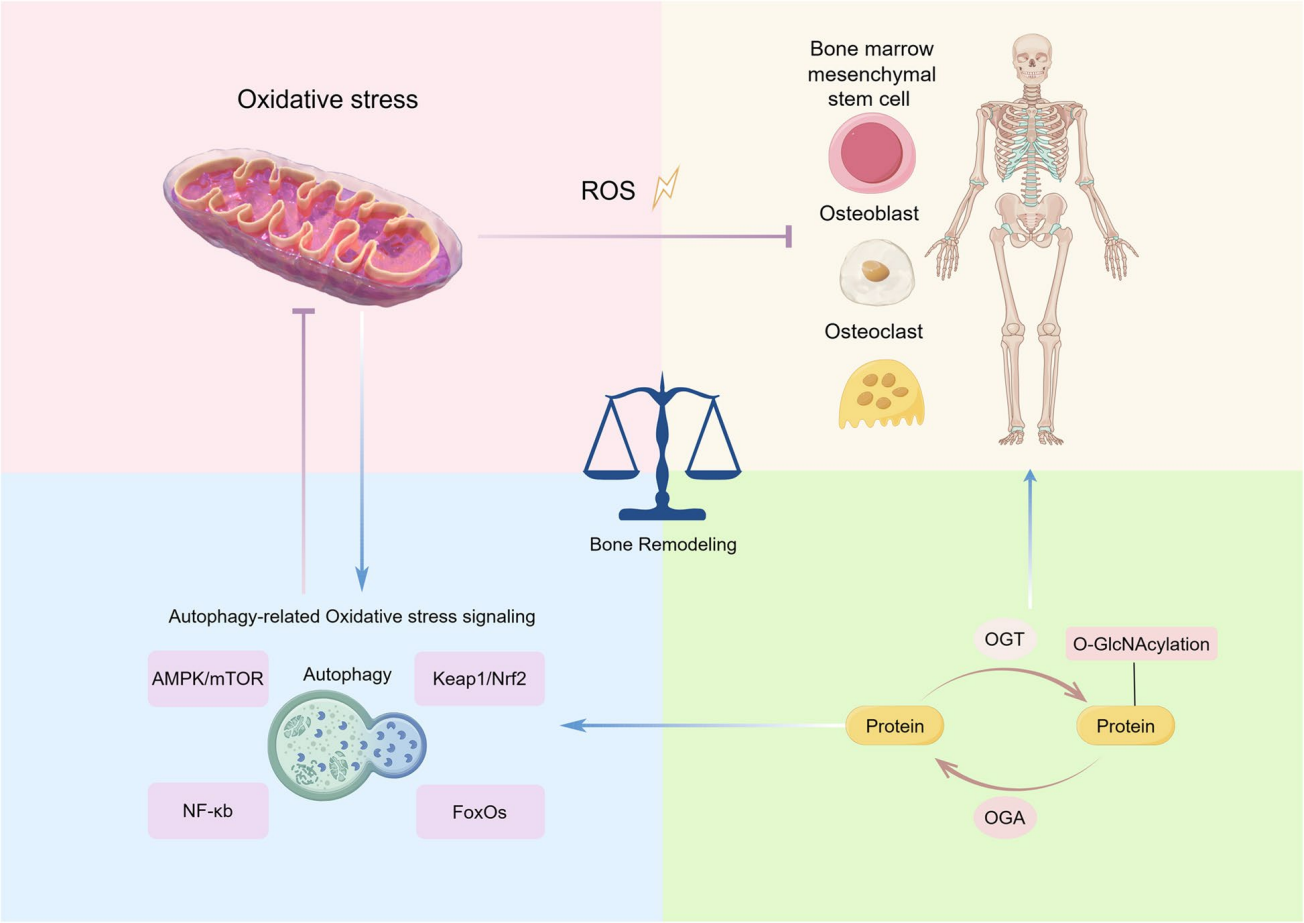

## Background

Oxidative stress results from an imbalance in cellular physiology between reactive oxygen species (ROS) and antioxidant signaling, wherein ROS production surpasses the capacity of the ROS defense system [1]. The mitochondrial respiratory chain is the primary endogenous source of ROS, and intracellular ROS is maintained at a consistently low level under normal physiological conditions [2]. This balance is not only involved in intracellular signaling regulation but also sustains bone metabolism [3].

Bones exhibit dynamic characteristics throughout life, including the resorption of aging or compromised bone tissue and the subsequent deposition of new bone material. These ongoing alterations are termed bone remodeling and are vital for maintaining the structural integrity of the skeletal system [4]. However, besides physiological ROS generation, pathophysiological factors such as hyperglycemia [5], medication-related osteonecrosis of the jaw (MRONJ) [6], and osteoporosis [7] can elevate ROS levels in bone tissues and disrupt the remodeling process.

The degradation and maintenance of intracellular proteins and organelles depend on the intricate autophagy process [8]. Cells sequester misfolded proteins or damaged organelles into lysosomes for degradation, thereby sustaining cell homeostasis during starvation, hypoxia, infection, and other stress [9, 10]. Autophagy dysfunction is correlated with various diseases, and its association with bone diseases has been extensively studied, especially in the metabolism of bone marrow-derived mesenchymal stem cells (BMSCs), osteoblasts, and osteoclasts [11].

O-linked N-acetylglucosamine protein modification (O-GlcNAcylation) is a monosaccharide post-translational modification (PTM), which is implicated in several physiological functions, such as metabolism, signaling, transcription, and translation [12]. As a crucial sensor of oxidative stress, many proteins involved in the autophagic process have been identified as targets for O-GlcNAcylation [13]. Only two conserved enzymes, O-GlcNAc transferase (OGT) and O-GlcNAcase (OGA), are involved in O-GlcNAcylation. OGT facilitates the attachment of O-GlcNAc to the hydroxy group of serine and threonine residues, whereas OGA hydrolytically cleaves O-GlcNAc from modified proteins [12].

The depletion of OGT in BMSCs was shown to compromise bone formation in mice, and osteoclast-specific OGT knockout increased bone mineral density, which established the essential role of O-GlcNAcylation in bone remodeling [14, 15]. Presently, most research on O-GlcNAcylation-mediated bone remodeling has concentrated on metabolic pathways encompassing glucose and glutamine uptake and utilization, with the precise phase and principal cellular localization of O-GlcNAcylation governing bone remodeling yet to be elucidated. Recent investigations introduced a novel perspective, indicating that OGT inhibition disrupts mitochondrial function, culminating in oxidative stress and autophagy [16]. Thus, as a crucial nexus linking signal transduction and metabolism, O-GlcNAcylation orchestrates oxidative stress and autophagy [17].

The objective of this review was to explore the potential mechanism by which O-GlcNAcylation mitigates oxidative stress in bone remodeling and the associated bone pathologies through autophagy. Additionally, novel strategies for improving bone remodeling in various diseases via O-GlcNAcylation are also provided.

## Interplay between O-GlcNAcylation and oxidative stress

Several pathological factors, including hyperglycemia and alterations in hormone levels, can induce oxidative stress, which is an autonomous risk factor for bone diseases. However, O-GlcNAcylation intricately connects cellular metabolic status with oxidative stress via the hexosamine biosynthetic pathway (HBP), thereby regulating bone remodeling.

### O-GlcNAcylation: a sensor of metabolic status

**Fig. 1:**
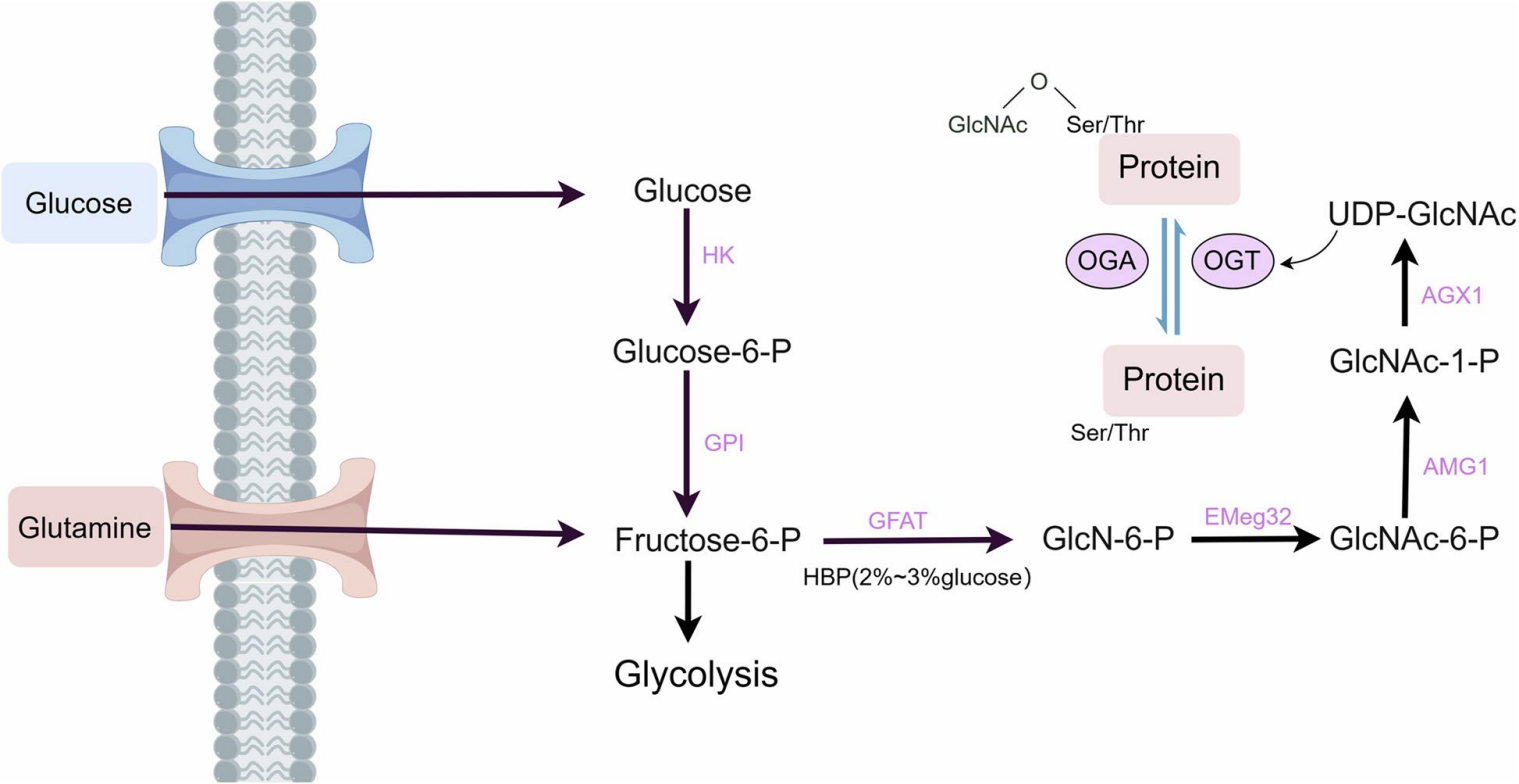
The hexosamine biosynthetic pathway and O-GlcNAcylation. Approximately 2% to 5% of glucose is directed toward the HBP during glycolysis. The pivotal stage in the HBP, governed by the enzyme GFAT, involves the conversion of fructose-6-phosphate to glucosamine-6-phosphate through glutamine and the involvement of enzymes, subsequently yielding UDP-GlcNAc. As a substrate for OGT, UDP-GlcNAc actively engages in the O-GlcNAcylation of cytoplasmic or nuclear proteins. O-GlcNAcylation equilibrium is maintained by OGT and OGA. Abbreviations: HK, hexokinase; GPI, glucose phosphate isomerase; Gluc-6P, glucosamine-6-phosphate; Fruc-6P, fructose-6-phosphate; GlcN-6P: glucosamine-6-phosphate; GlcNAc-6P: N-acetylglucosamine-6-phosphate; GlcNAc-1P, N-acetylglucosamine-1-phosphate; GAFT, glutamine-fructose-6-phosphatase aminotransferase; EMeg32, glucosamine-6-phosphate acetyl-transferase; AGM1, phosphoacetylglucosamine mutase

Unlike traditional glycosylation, O-GlcNAcylation adds an O-GlcNAc moiety to nucleocytoplasmic and mitochondrial compartments, maintaining it as a monosaccharide rather than further developing into a polysaccharide [18]. About 2–5% of total cellular glucose is funneled through the HBP, which is a glycolysis shunt that is activated by enhanced glucose absorption [19]. The HBP produces uridine-5′-diphosphate-N-acetylglucosamine (UDP-GlcNAc), the donor substrate for OGT [20] (Fig. 1). O-GlcNAcylation also interacts with other PTMs like phosphorylation and ubiquitination to affect signal transduction in stressful environments, which further influences disease outcomes and prognoses [21–23]. Therefore, O-GlcNAcylation serves as a nutrition sensor, tying the metabolic state to the control of several signaling cascades [24]. The absence of either OGT or OGA in mice resulted in embryonic and perinatal lethality, underscoring their crucial roles in survival and development [25].

### Oxidative stress and bone remodeling

The equilibrium of the bone remodeling processes is crucially influenced by oxidative stress [26]. Aged mice exhibited up-regulated oxidative stress genes and down-regulated osteoblast-related genes compared to young mice, potentially contributing to age-related osteoporosis [27]. Additionally, excessive ROS produced by oxidative stress promotes osteoclast differentiation. Antioxidant drugs have demonstrated their efficacy in treating osteoporosis by enhancing osteoblast and bone development while inhibiting osteoclast and bone resorption [28]. In contrast, reducing nuclear factor erythroid 2‐related factor 2 (Nrf2)-responsive antioxidants were shown to increase receptor activator of NF-κB ligand (RANKL)-induced intracellular ROS levels, contributing to osteoclast differentiation [29].

### O-GlcNAcylation and oxidative stress

The interplay of O-GlcNAcylation and protein oxidation is essential in regulating cellular damage [30, 31]. O-GlcNAcylation levels can respond to oxidative damage through signal transduction and metabolism [32]. For instance, oxidative stress increases the O-GlcNAcylation of nucleoplasmic proteins, whereas suppressing O-GlcNAcylation increases cellular vulnerability to stress, resulting in reduced cell viability and bone formation [33, 34]. Previous studies showed that this may be related to the increased activity of signal transducer and activator of transcription(STAT3) and forkhead box transcription factor O1 (FoxO1) mediated by OGT [35].

O-GlcNAcylation can also change intracellular ROS levels and influence bone remodeling. Glucose-6-phosphate dehydrogenase (G6PDH) was reported to be activated by hyper O-GlcNAcylation, thereby up-regulating NADPH/NADP+ and GSH/GSSG couples to relieve oxidative stress [36]. A high GSH/GSSG ratio also significantly increased the mRNA expression levels of markers associated with osteogenic differentiation and mineralization [37]. Similarly, the nuclear factor of activated T cells (NFAT) family and NF-κB activities were suppressed by elevated O-GlcNAcylation, which inhibited RANKL-induced osteoclast differentiation [38, 39].

Although some oxidative stress-related O-GlcNAcylation substrates have been identified, more work is needed to determine the complete O-GlcNAcylated site that transduces these signals in mitochondrial network homeostasis [40]. A thorough comprehension of how O-GlcNAcylation contributes to signal transduction under oxidative stress conditions could reveal novel avenues for therapeutic interventions in bone remodeling.

## O-GlcNAcylation: a sweet regulator of autophagy

Autophagy functions as a cellular self-defense mechanism that shields cells from metabolic stress and oxidative damage. Recent studies showed that O-GlcNAcylation exerted varied effects on proteins engaged in distinct phases of autophagy, which affected the overall process of bone remodeling by regulating autophagy in BMSCs, osteoclasts, and osteoblasts [13, 41].

### Autophagy and bone remodeling

A previous study proposed a dual association between autophagy and BMSCs, whereby the regulation of autophagy affects the function of BMSCs, and BMSCs may also modulate the autophagy of immune cells, ultimately influencing the therapeutic properties of BMSCs [42]. For example, macrophages with low-inflammatory characteristics can induce autophagy in BMSCs, thereby enhancing osteogenesis [43]. Anti-inflammatory macrophages frequently exhibit increased levels of O-GlcNAcylation and OGT. Further investigations are needed to ascertain its potential correlation with autophagy [44]. Additionally, increased mitochondrial respiration with ATP production is vital for BMSCs or pre-osteoblast differentiation and leads to further increases in endogenous ROS [45]. However, as a critical form of selective autophagy, OGT-regulated mitophagy selectively removes dysfunctional or redundant mitochondria to ensure mitochondrial quality, which is essential for preventing BMSCs senescence [46–48].

Compared to cytoprotection in osteoblasts, autophagy actively promotes osteoclast differentiation. A recent study demonstrated that metformin mitigated bone loss by inhibiting osteoclast differentiation, a consequence of dysregulated autophagy in osteoclast precursors [49]. This phenomenon may be attributed to the capacity of metformin to reduce the O-GlcNAcylation of AMP-activated protein kinase (AMPK), thus enhancing cell cycle arrest [50]. Additionally, the ablation of autophagy in osteoblasts promoted bone resorption by triggering the release of RANKL [51]. These studies underscore the role of autophagy as a target of O-GlcNAcylation during bone remodeling.

### Autophagy mechanisms

**Fig. 2:**
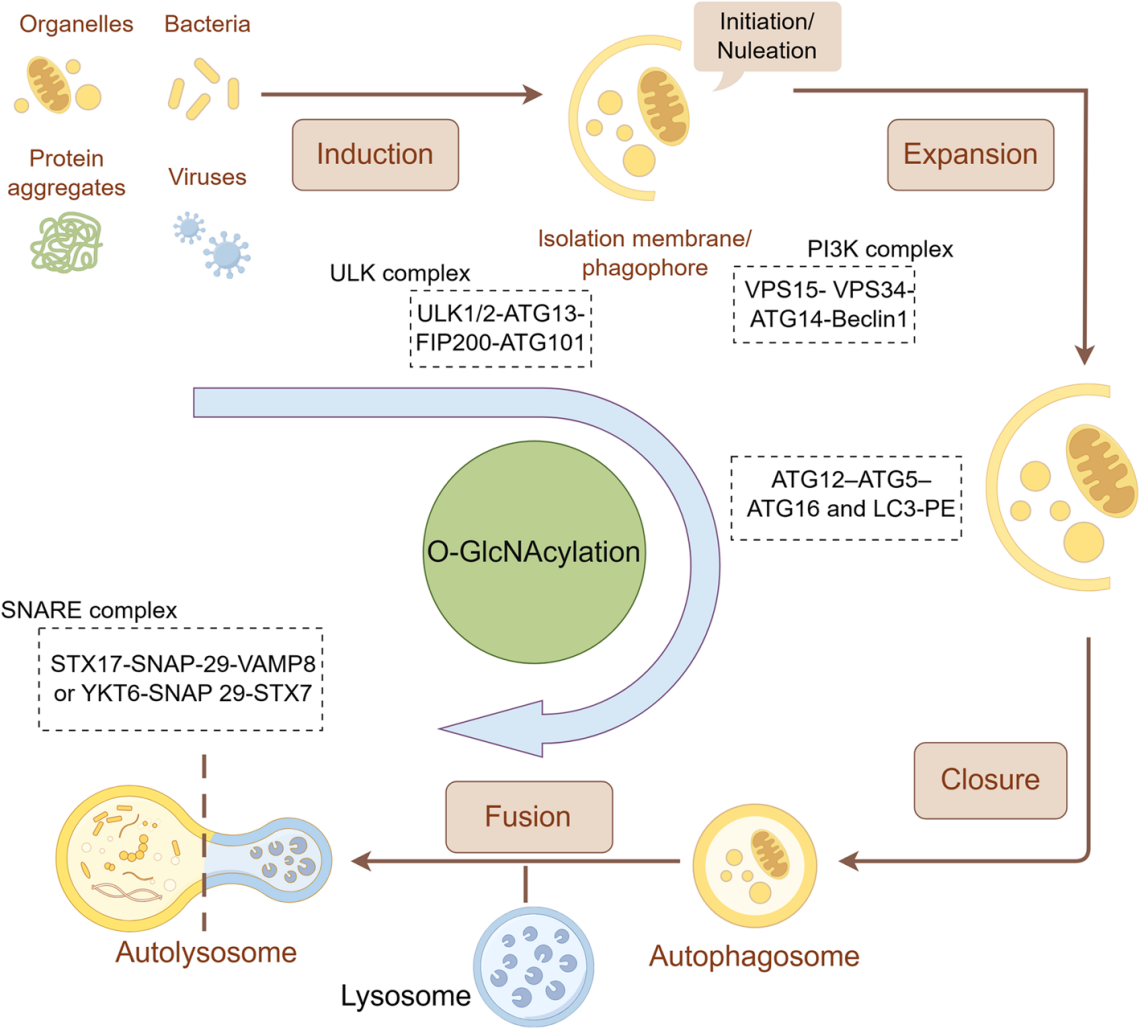
Brief representation of the autophagy process. The process of autophagy comprises initiation, nucleation, phagophore expansion and autolysosome fusion. Each process is regulated by many autophagy-related proteins and genes, such as the ULK1 complex, PI3K complex, LC3, and the SNARE complex, which are targets of O-GlcNAcylation

The development of the isolation membrane, formerly known as the phagophore, marks the initiation of autophagy. The extension of this membrane is further facilitated by autophagy-related (ATG) genes and proteins, culminating in the formation of autophagosomes. Subsequently, the autophagosome merges with the endosomal lysosomal apparatus, which converts it to an autolysosome to degrade isolated cytoplasmic materials [52]. The entire autophagy process primarily encompasses the formation of autophagosomes (initiation, nucleation, and phagophore expansion) and autolysosomes [53] (Fig. 2). The molecular regulation of autophagy is as follows:

i) **Initiation:** FAK family kinase-interacting protein of 200 kDa (FIP200), ATG13, ATG101, and ULK1/2 make up the UNC51-like kinase (ULK) complex [54]. ULK1 and ULK2 phosphorylate FIP200 and ATG13 to initiate the phagophore production process [55].
ii) **Nucleation:** A class III PI3K complex made up of Beclin1, ATG14, vacuolar protein-sorting 15 (VPS15), and VPS34 is the target of the activated ULK complex. Phosphatidylinositol 3-phosphate (PI3P), which VPS34 generates on phagophore membranes, facilitates the recruitment of additional autophagic machinery protein complexes [56]. Recent findings indicated that ATG14 also enhanced autophagosome fusion with the endolysosomal compartment [57].
iii) **Expansion:** The lipidation of microtubule-associated protein 1 light chain 3 (MAP1LC3; also known as LC3) with phosphatidylethanolamine is the result of the recruitment of ATG5-ATG12-ATG16 complex to the autophagosome membrane [8]. LC3 is required for expansion, and ATG4B is essential for LC3 lipidation and processing [58].
iv) **Formation of autolysosomes:** RAB GTPases, tethering proteins, and soluble N-ethylmaleimide-sensitive factor attachment protein receptors (SNAREs) work in concert to facilitate the merging of autophagosomes with late endosomes/lysosomes. Several SNARE proteins primarily mediate this process: STX17 and SNAP29 on autophagosomes and VAMP8 on late endosomes/lysosomes or YKT6 and SNAP29 on autophagosomes and STX7 on late endosomes/lysosomes. Recent studies also identified multiple tethered proteins facilitating the fusion process, including the HOPS complex, EPG5, and PLEKHM1 [59].

Besides the essential ATG proteins, specific adaptors for autophagy, such as SQSTM1 (p62), NBR1, and NDP52, play crucial roles in selectively recruiting aberrant proteins to autophagosomes and promoting their subsequent fusion with lysosomes for degradation [60].

### Regulation of O-GlcNAcylation on autophagy

O-GlcNAcylation can promote the formation of autophagosomes while inhibiting autolysosome formation. Thus, identifying effective targets for alleviating oxidative stress by promoting autophagy is predicted to facilitate bone remodeling. The consequences of O-GlcNAcylation on bone remodeling through autophagy under different O-GlcNAcylation-related stimuli and the possible mechanisms are summarized in Table 1.

**Table 1:** The effect of O-GlcNAcylation-related stimuli targeting autophagy on bone remodeling

Stress factor	Target	Effect on autophagy	Cell type/model	Effect on bone remodeling	Ref
Starvation	ULK1	Activation	H1299, HEK293, and SW620 cells	Initiated phagophore formation and improved bone regeneration by promoting osteogenic differentiation	[61, 62]
Starvation	ATG4B	Inhibition	SH-SY5Y cells	Inhibited osteoclast maturation	[63–65]
Diabetes	Beclin 1	Inhibition	Mouse cardiomyocytes	Reduced the mineralization activity of osteoblasts and impaired osteoclast function	[66, 67]
Low expression of OGT	SNAP-29	Activation	A2780 and SKOV3 cells	Contributed to osteoblast function and impaired osteoclast differentiation	[68, 69]
Overexpression of OGT	ATG5	Inhibition	*Drosophila melanogaster*	Reduced osteoblast and osteoclast differentiation	[70, 71]

#### Regulation of O-GlcNAcylation in autophagosome formation

The necessity of O-GlcNAcylation for autophagy has been demonstrated using an OGT knockout mouse model, which found that the absence of OGT reduced autophagy levels [72]. Further investigation revealed that ULK1 underwent O-GlcNAcylation at the threonine 754 site under glucose shortage conditions, which was essential for activating lipid kinase VPS34 and producing of PI3P [61]. The AMPK-ULK1-autophagy axis has been shown to promote osteoblast differentiation and bone regeneration, suggesting the potential therapeutic effect of O-GlcNAcylation on bone remodeling [62]. Beclin-1, an essential contributor to both autophagy and osteoclast formation, is O-GlcNAcylated, thereby inhibiting autophagy. This process leads to defects in chondrocyte differentiation and osteoclast dysfunction [66, 67]. Additionally, the O-GlcNAcylation of ATG4B was shown to up-regulate its proteolytic activity to promote autophagy, whereas the O-GlcNAcylation ATG5 suppressed autophagy [65, 70]. Whether O-GlcNAcylation can amplify the maturation of osteoclasts caused by the activation of ATG4-LC3 deserves further study [64].

#### Regulation of O-GlcNAcylation in autolysosome formation

O-GlcNAcylation appears to have an inhibitory effect in the fusion stage of autophagosomes and lysosomes, contrary to its role in the initial stage [73]. A decrease in the OGT-induced O-GlcNAcylation of SNAP-29 caused the SNARE complex to assemble more readily, which enhanced cisplatin-induced autophagy [68]. In contrast, augmenting SNAP29 O-GlcNAcylation inhibited the formation of the SNARE fusion complex, leading to increased ROS generation and subsequent apoptosis [69]. The increase in ROS not only promoted the differentiation of osteoclasts but also hampered the assembly of the SNARE complex due to SNAP O-GlcNAcylation, which, in turn, inhibited the production of bone matrix mediated by osteoblasts [74].

## O-GlcNAcylation: at the crossroads of oxidative stress and autophagy

Although oxidative stress and autophagy have been well-investigated, the impact of O-GlcNAcylation on both processes varies in different bone remodeling conditions, possibly due to the collective influence of O-GlcNAcylation across diverse signal transduction pathways. Consequently, we have consolidated the insight into autophagy-related oxidative stress signaling pathways, such as ROS/AMPK/mTOR, ROS/Keap1/Nrf2, ROS/FoxOs, and ROS/NF-κB, hoping to elucidate the effects of O-GlcNAcylation on bone remodeling under oxidative stress.

### AMPK/mTOR

#### AMPK/mTOR signaling in oxidative stress

**Fig. 3:**
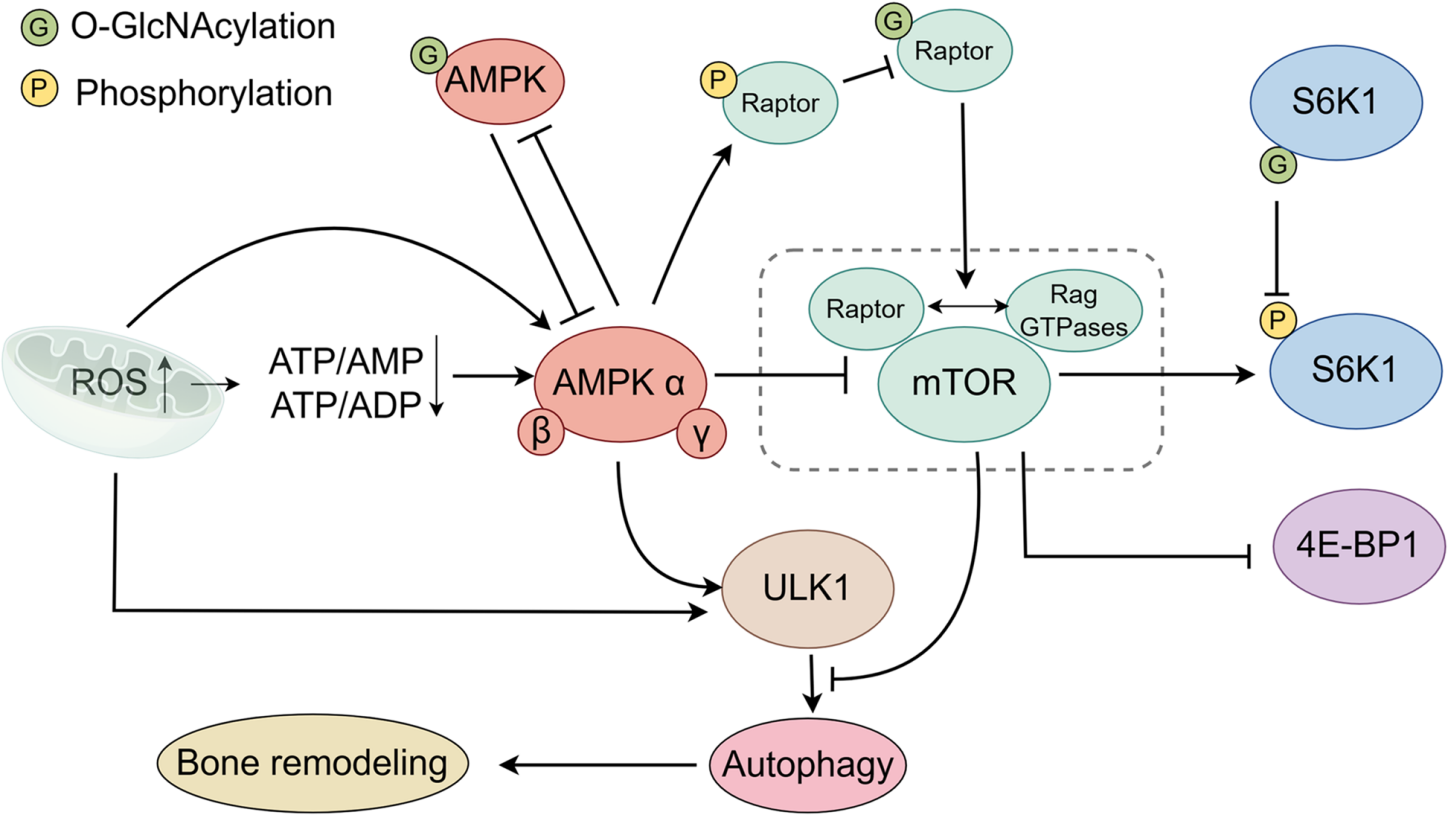
ROS/AMPK/mTOR signaling pathways and O-GlcNAcylation. ROS influences AMPK activity through direct interactions or indirectly by modifying adenine nucleotide ratios. AMPK, in turn, can either activate ULK1 or inhibit mTORC1, promoting autophagy as a response to mitigate oxidative stress. The O-GlcNAcylation of Raptor activates the mTORC1 complex, thereby impacting downstream targets such as S6K1 and 4EBP1, both of which are subject to O-GlcNAcylation-mediated regulation

AMPK consists of regulatory β- and γ-subunits and catalytic α-subunits, and is activated by intracellular ATP synthesis and elevated ROS levels [75, 76]. The mammalian target of rapamycin (mTOR) serves as the catalytic subunit within two separate entities identified as mTORC1 and mTORC2 [77]. mTORC1 acts as a major regulator of protein synthesis by regulating eukaryotic initiation factor binding protein 1 (4E-BP1) and p70 ribosomal protein S6 kinase 1(S6K1). Additionally, mTORC1 can suppress autophagy by regulating the ULK1 complex. mTORC2 also functions as a crucial upstream regulator that initiates the PI3K-AKT pathway [78] (Fig. 3).

Early studies found that exogenous H2O2 could activate AMPK directly or by reducing the ATP/ADP ratio [79, 80]. Conversely, elevated glucose levels promote ROS generation, which causes the ubiquitination of AMPKα and subsequent degradation [81]. These contrasting impacts imply that the multiple regulatory effects of ROS on AMPK/mTOR signaling are closely related to glucose metabolism. Recent studies found that AMPK activation could inhibit ROS-induced osteoclastogenesis. Thus, the role of O-GlcNAcylation in this process deserves further investigation [82].

#### ROS/AMPK/mTOR/autophagy signaling

The association between oxidative stress and AMPK/mTOR is frequently mediated by autophagy. ULK1 activation can be achieved through the direct phosphorylation of AMPK or indirectly by inhibiting mTORC1, thus alleviating its suppressive influence on ULK1 [83, 84]. BMSC-derived exosomes were reported to inhibit ROS/NLRP3 inflammasomes through AMPK/mTOR/autophagy signaling [85], and AMPK/mTOR/ULK-1 signaling has been shown to ameliorate rheumatoid arthritis [86]. Thus, the ROS/AMPK/mTOR/autophagy signaling pathway has emerged as a promising therapeutic target in bone remodeling [87].

#### O-GlcNAcylation of ROS/AMPK/mTOR/autophagy signaling

As the key regulator of the mTORC1 complex, AMPK can also be O-GlcNAcylated [88, 89]. Nutrient-sensitive pathways regulate both O-GlcNAcylation and AMPK, linking metabolic homeostasis to the control of various intracellular processes [90]. The activity of AMPK is blocked after O-GlcNAcylation, which then suppresses autophagy and ULK1 activity [91]. Notably, ULK1 can be activated after O-GlcNAcylation and promote bone remodeling. Whether the O-GlcNAcylation of AMPK counteracts this process has not been reported [61, 62]. In the absence of O-GlcNAcylation, AMPK activation can facilitate skeletal muscle to utilize glucose more efficiently [92]. However, the natural antioxidant α-lipoic acid was shown to decrease interactions between AMPK and OGT in diabetic mice, further contributing to cell survival, partly through AMPK activation or OGT inhibition [93]. In summary, increased O-GlcNAcylation significantly decreased autophagy through AMPK/mTOR signaling, indicating the inhibitory impact of AMPK O-GlcNAcylation on bone remodeling.

In addition to the AMPK pathway, direct cross-regulations between mTOR signaling and O-GlcNAcylation have also been reported [94]. mTOR O-GlcNAcylation inhibition increased autophagosome numbers and the levels of the autophagic markers SQSTM1/p62 and LC3-II [95]. The treatment of HepG2 cells with mTOR inhibitors to induce autophagic flux resulted in an unanticipated reduction in overall O-GlcNAcylation due to decreased OGT and increased OGA [96]. Conversely, increased levels of O-GlcNAcylation were observed after increasing mTOR activation [97]. Recent studies suggested that the regulation of mTORC1 activation by glucose is tightly controlled by the O-GlcNAcylation of Raptor, a crucial constituent of mTORC1. Specifically, O-GlcNAcylation occurs at threonine 700 of Raptor, facilitating its binding with Rag GTPases. This binding facilitates the relocation of mTOR to lysosomal surfaces, initiating mTORC1 activation. Raptor O-GlcNAcylation was suppressed by the AMPK phosphorylation of Raptor, which prevented Raptor from interacting with Rags GTPases [98]. The RhoA protein of GTPases contributes to the differentiation of osteoclasts and ultimately causes osteoporosis, indicating that O-GlcNAcylation can not only inhibit autophagy by activating mTOR in bone remodeling but also promote osteoclast absorption [99].

The downstream mTOR pathway also responds to O-GlcNAcylation [100]. OGT was reported to inhibit the proinflammatory activation of macrophages by catalyzing S6K1 O-GlcNAcylation and, hence, inhibit mTORC1 signaling [100], suggesting that a comparable mechanism may weaken bone absorption caused by osteoclasts in oxidative stress.

In general, AMPK negatively regulates the mTOR pathway to promote autophagy and alleviate oxidative stress, while the O-GlcNAcylation of AMPK reduces its activity. O-GlcNAcylation not only directly regulates mTORC1 but also influences the downstream mTOR pathway. The regulation of O-GlcNAcylation in the ROS/AMPK/mTOR/autophagy pathway integrates nutritional signals to respond to oxidative stress, which further promotes bone remodeling.

### Keap1/Nrf2

#### Keap1/Nrf2 signaling in oxidative stress

**Fig. 4:**
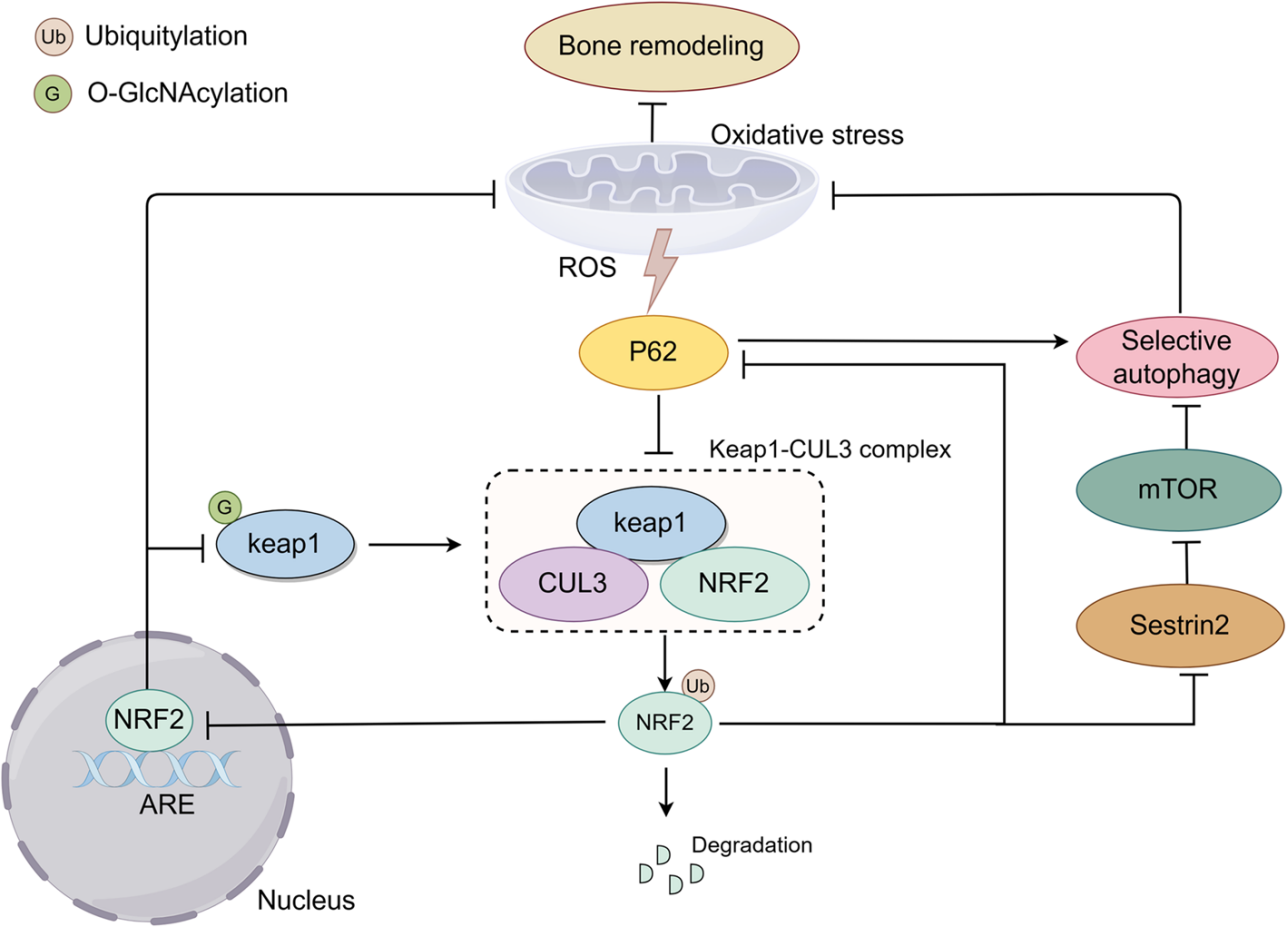
p62-Keap1-Nrf2 feedback loop and O-GlcNAcylation. O-GlcNAcylation is necessary for Nrf2 degradation by Keap1-CUL3 ubiquitin E3 ligase. Keap1-CUL3 ubiquitin E3 ligase functionality decreases when exposed to oxidative stress, resulting in the translocation of Nrf2 into the nucleus and initiating a cascade of antioxidant gene activation. At the same time, activated p62 can promote the generation of Nrf2 through autophagy to form a positive cycle

Nrf2 is an antioxidative transcription factor, safeguarding cells from oxidative stress by controlling antioxidant response elements (ARE) [101] (Fig. 4). Kelch‐like ECH‐associated protein 1 (Keap1) develops a ubiquitin E3 ligase complex with CULLIN3 (CUL3) under quiescent conditions, which results in the ubiquitination and degradation of Nrf2 [102]. However, ubiquitin E3 ligase activity is decreased when Keap1 is subjected to oxidative stress, which ultimately facilitates the subsequent translocation of Nrf2 to the nucleus, initiating the antioxidant response [103]. Recent findings indicated that osteocyte Nrf2 activity promoted the expression of the osteocytic gene and was necessary for bone homeostasis [104]. An Nrf2 activator was shown to inhibit osteoclast formation and bone resorption in vivo by interfering with NF-κB signaling transduction [105]. Conversely, osteoclast differentiation was enhanced after attenuating the Nrf2-mediated antioxidant response [29].

#### ROS/Keap1/Nrf2/autophagy signaling

P62 is a selective autophagy receptor protein that bridges between autophagy and the Keap1-Nrf2 system [106–108]. The pivotal function of p62 involves delivering diverse ubiquitination cargo to the autophagosome, culminating in its degradation by lysosomes, which is further stimulated by oxidative stress [108, 109]. p62 can form a complex with Keap1 (p62-Keap1 complex) that undergoes elimination via the ubiquitin–proteasome system or induces the degradation of Keap1 via selective autophagy [107, 110]. Additionally, Nrf2 induces the expression of p62 and Sestrin2 in a reciprocating manner, which establishes a positive feedback loop between p62 and Keap1-Nrf2 that amplifies protective effects on cells [110, 111]. Simultaneously, Sestrin2 activates autophagy by inhibiting mTORC1 expression [112]. Recent studies showed that phosphorylating p62 at Ser349 and Thr269/Ser272 protected bones from destruction, ameliorating arthritis by activating the p62-Keap1-Nrf2 feedback loop [113]. Thus, preserving bone homeostasis depends on the balance of a positive feedback loop that includes p62, Keap1, and Nrf2.

#### O-GlcNAcylation of ROS/Keap1/Nrf2/autophagy signaling

O‐GlcNAcylation is necessary for the efficient ubiquitination of Nrf2 since O‐GlcNAcylation of Keap1 at serine 104 was shown to enhance its productive interaction with CUL3 [114]. Increasing Keap1 O-GlcNAcylation led to the ubiquitination and degradation of Nrf2 while simultaneously inhibiting autophagy [115]. In contrast, ovarian cancer cells with low‐OGT activity tended to have strong Nrf2 activation signatures [116]. A previous study showed that Nrf2 activation could inhibit oxidative stress in ovariectomized rats, preventing osteoporosis and promoting osteogenesis [117]. O-GlcNAcylation inhibits bone remodeling in Keap1-Nrf2 signal transduction, which involves inhibiting the antioxidant effect of Nrf2 and further blocking the amplification of autophagy by the p62-Keap1-Nrf2 feedback loop [118]. Conversely, as a member of the same Nrf2 family, OGT improves the stability of the Nrf1a protein [119]. In addition, CUL3 can down-regulate OGT expression, which depends on binding to the OGT promoter region [120].

As recently as 30 years ago, studies found that p62 activity was sensitive to glucose metabolism and was modified by O-GlcNAc after translation [121, 122]. Although the p62 site of O-GlcNAcylation has been identified, its downstream effects remain unknown [123]. The protective role of the p62/Keap1/Nrf2 feedback loop in bone destruction has been proven, suggesting that the O-GlcNAcylation of p62 is a potential therapeutic target in bone remodeling [113].

The p62-Keap1-Nrf2 positive feedback loop connects Nrf2 to autophagy, alleviating ROS exposure and promoting bone remodeling. O-GlcNAcylation acts as a terminator and destroys the positive effects on bone remodeling. Consequently, it is essential to manage interactions between the Keap1-Nrf2 pathway, autophagy, and O-GlcNAcylation for effective bone remodeling.

### FoxOs

#### FoxOs in oxidative stress

**Fig. 5:**
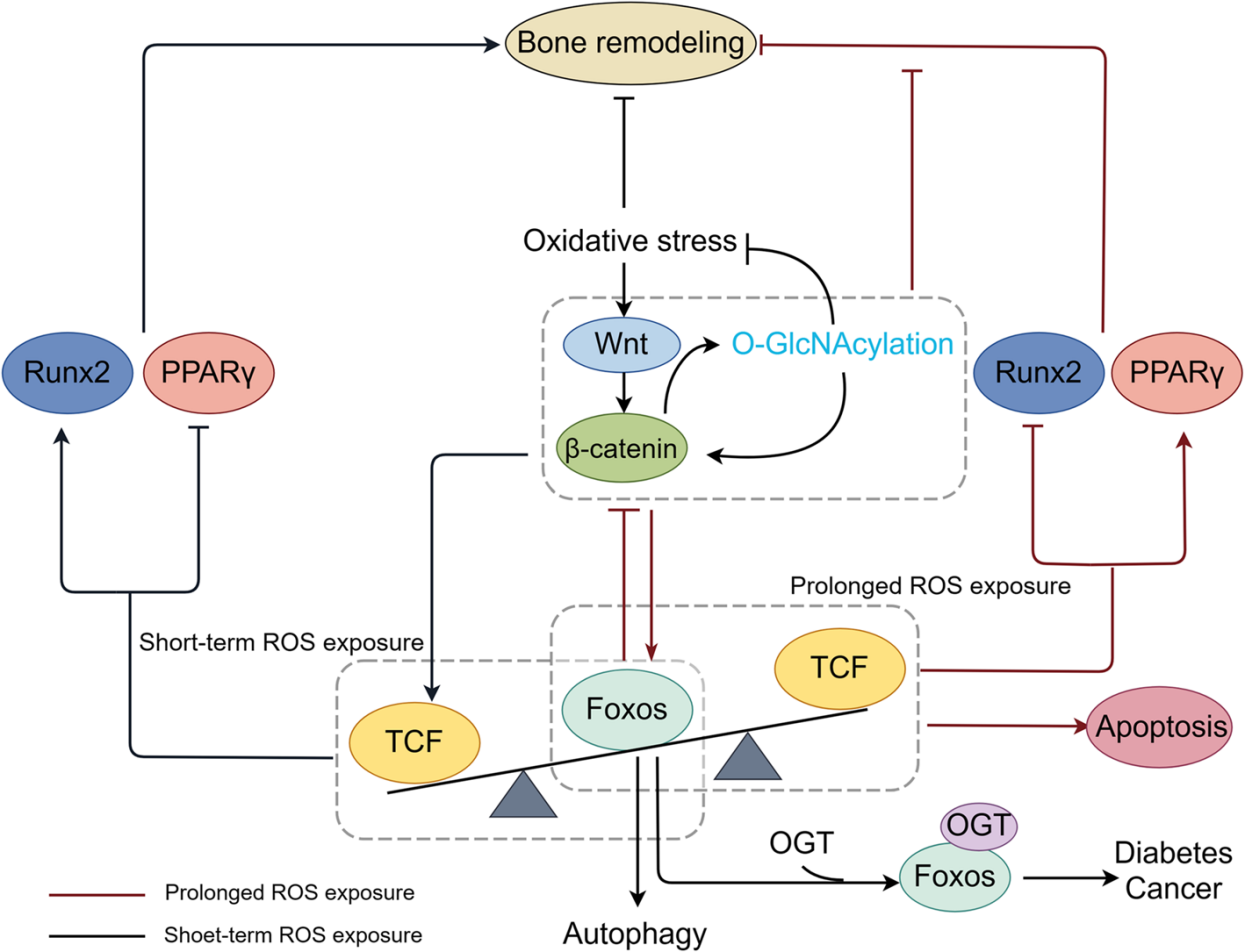
O-GlcNAcylation response to FoxO and autophagy in oxidative stress. Upon exposure to ROS, β-catenin engages with FoxO, triggering the activation of antioxidant genes and the induction of autophagy to mitigate oxidative stress. Prolonged ROS exposure disrupts the interaction between TCF and β-catenin, promoting apoptosis and decreasing osteogenic differentiation. O-GlcNAcylation emerges as a mechanism capable of enhancing β-catenin expression, counteracting the detrimental consequences of persistent ROS stimulation. However, both OGT and FoxO genes are implicated in the onset of diabetes and cancer

FoxO transcription factors, namely FoxO1, FoxO3, FoxO4, and FoxO6, regulate antioxidant responses in glucose metabolism [124, 125]. Runx2 and PPARγ are the main transcription factors for osteogenesis and adipogenesis, respectively, and are controlled by Wnt/β-catenin through binding to T cell factor (TCF). Wnt/β-catenin significantly contributes to the osteogenic differentiation of BMSCs under normal circumstances [126]. However, as modulators of the cellular stress response, FoxOs are stimulated by ROS to interact with β-catenin, thereby jointly activating the transcription of target genes related to the antioxidant response [127]. In contrast, FoxO activity is influenced by ROS and is associated with post-translational changes, such as phosphorylation and O-GlcNAcylation [128–130] (Fig. 5).

Considering the bidirectional regulation between FoxOs and ROS, FoxOs assume a dual role in bone remodeling under oxidative stress. The impaired functionality of FoxOs in bone cells contributes to osteoarthritis, osteoporosis, and other bone diseases, with FoxO1 and FoxO3 being extensively expressed and studied [131]. The overexpression of FoxO3 in osteoclasts augments bone mass by stimulating catalase to inhibit ROS and bone resorption [132, 133]. FoxO1 promotes the differentiation of BMSCs into osteoblasts by suppressing the transcription and expression of PPARG/PPARγ, which adversely regulates adipogenesis [134]. However, prolonged ROS exposure was shown to facilitate the redirection of β-catenin from TCF to FoxO transcription, consequently hindering the Wnt/β-catenin signaling pathway and diminishing osteogenic functionality [133]. Recent studies found that the down-regulation of FoxO1 expression could prevent BMSC apoptosis in acute inflammation [135], and the absence of FoxO1 was reported to indirectly enhance bone remodeling and mitigate age-related bone resorption by improving the binding of β-catenin and TCF [136]. Notably, the sustained activation of FoxOs increased PPARγ levels, forming complexes with β‐catenin and inducing β‐catenin degradation. Thus, the repressive action of prolong ROS can cause age-related decreases in bone mineral density during bone remodeling [131].

#### ROS/FoxOs/autophagy signaling

FoxO transcription factors, especially FoxO3 and FoxO1, can also mediate autophagy activity to regulate bone remodeling under oxidative stress [41]. An increase in ROS was reported to activate FoxO3 phosphorylation at Ser294 and contribute to its nuclear translocation, ultimately mitigating ROS levels by up-regulating autophagy [137]. Similar protective effects were observed in osteoarthritis-induced meniscus injury alleviation [138]. However, the FoxO3-mediated transcriptional activation of autophagy-related genes, such as LC3, BNIP3, Beclin-1, ATG4, and ATG12, was inhibited after Akt down-regulated FoxO3 expression in starvation [139]. Additionally, early-onset osteoarthritis was observed in FoxO1 or FoxO3-deficient mice, whereas FoxO1 shielded chondrocytes from oxidative stress and promoted the overexpression of genes related to autophagy and proteoglycan 4, a crucial joint lubricant [140].

#### O-GlcNAcylation of ROS/FoxOs/autophagy signaling

Given the crucial involvement of FoxOs in glucose metabolism, it is reasonable to contemplate their interaction with O-GlcNAcylation [141, 142]. Since 1991, studies have hinted that elevated O-GlcNAcylation levels might stimulate insulin resistance [143, 144]. The increased O-GlcNAcylation of FoxO1 was observed in diabetes, resulting in the activation of FoxO1 and regulating antioxidative stress in response to glucose metabolism [145]. Recent findings that identified OGT-FoxO1/3 gene fusion in multiple tumors suggest that the O-GlcNAc/FoxOs axis may be the basis for these observations [146, 147]. Another study observed the nuclear translocation of FoxO1 and increased autophagy flux in a hyperglycemic environment [148]. However, FoxO1, FoxO3, and FoxO4 knockout reduced the number of osteoclasts and improved bone remodeling in diabetes-induced osteoporosis [149].

Although these findings suggest that O-GlcNAcylation plays a positive role in antioxidation and autophagy in bone remodeling through FoxOs, their comprehensive mechanisms have not been fully explained. With the emergence of new research methods, the complex role of O-GlcNAcylation and β-catenin has been further revealed in recent years. By employing dual-specificity aptamers targeting OGT/β-catenin, O-GlcNAcylation was found to stabilize β-catenin [150]. O-GlcNAc elevation not only amplified β-catenin protein levels but also augmented its nuclear accumulation, which, in turn, increased O-GlcNAc expression levels [151]. However, the depletion of OGT decreased Wnt/β-catenin signaling activity during embryonic neurogenesis [152]. This phenomenon may be attributed to the direct competition between O-GlcNAcylation and phosphorylation at threonine 41 of β-catenin [153]. In addition, the Wnt/β-catenin signaling pathway can regulate glycolysis to promote the osteogenic differentiation of BMSCs and improve alveolar bone repair [154]. In summary, the current research indicates that O-GlcNAcylation plays a coordinating role in the FoxO signaling pathway. Under short-term ROS stimulation, O-GlcNAcylation promotes bone remodeling through FoxO-mediated antioxidation and autophagy, while under long-term ROS stimulation, it may counteract the adverse effects on bone remodeling by stabilizing β-catenin.

### NF-κB

#### NF-κB in oxidative stress

**Fig. 6:**
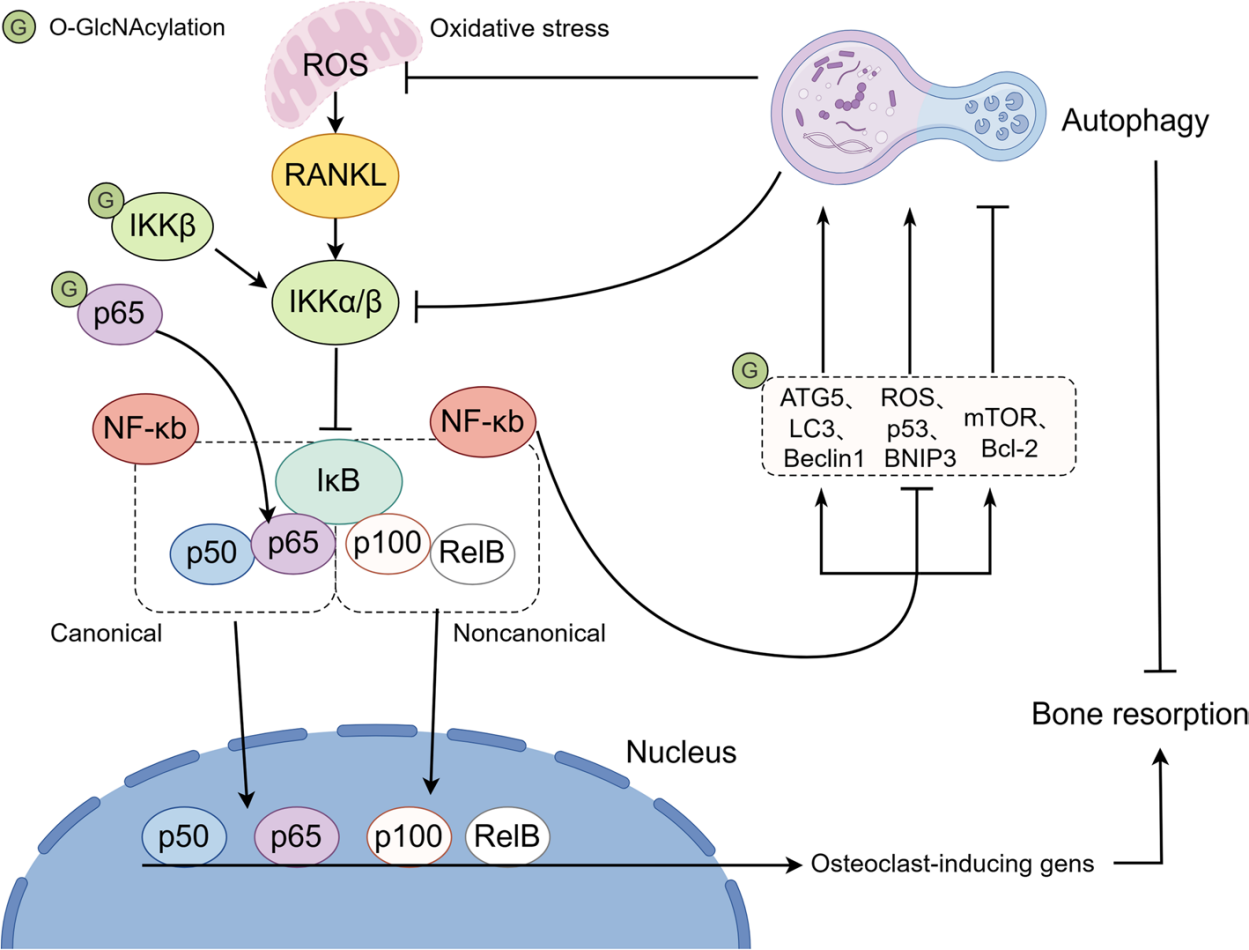
The involvement of O-GlcNAcylation and NF-κB in autophagy and oxidative stress in bone remodeling. ROS induces the degeneration of Iκb, enhancing the nuclear translocation of NF-κB p65/p50, consequently promoting osteoclast differentiation. Autophagy is regulated by the IKK/NF-κB signaling axis in a context-dependent manner influenced by stimuli and the environment. The O-GlcNAcylation of IKKβ and p65 can facilitate the activation of NF-κB

RelA (also called p65), c-Rel, RelB, NF-κB1 (which consists of p50 and its predecessor p105), and NF-κB2 (which consists of p52 and its precursor p100) are members of the NF-κB transcription factor family [155]. In many studies, NF-κB is commonly referred to as the p65/p50 heterodimer in the canonical NF-κB pathway, while RelB/p52 heterodimers are known to activate noncanonical signaling [156]. Notably, the RANKL-RANK-osteoprotegerin(OPG) system significantly influences bone remodeling. RANKL facilitates osteoclast differentiation, whereas OPG prevents osteoclast differentiation by inhibiting RANKL from binding to RANK [157]. IκBs, which are NF-κB inhibitory proteins, sequester p65/p50 heterodimeric complexes in the cytoplasm of unstimulated cells [155, 158]. The IKK complex (IKKα, IKKβ, and IKKγ) phosphorylates IκBs in response to RANKL stimulation, which causes the degradation of IκBs. As a result, the released NF-κB p65/p50 dimers undergo phosphorylation, facilitating nuclear translocation where they orchestrate the transcriptional regulation of downstream target genes crucial for osteoclast differentiation [159] (Fig. 6).

ROS activates NF-κB signaling and induces pro-inflammatory cytokines and chemokines [160]. Notably, ROS generated from purine nucleotide catabolism prompts the synthesis of RANKL in osteoblasts, fostering osteoclastogenesis, a recognized risk factor for osteoporosis [161]. Therefore, developing drugs that inhibit the NF-κB signaling pathway is of great significance for suppressing osteoclastogenesis and promoting osteogenesis [162]. For example, melatonin was shown to increase cartilage matrix formation and inhibit chondrocyte apoptosis by modulating the NF-κB signaling pathways to repair antioxidant defense system damage [163].

#### ROS/NF-κB/autophagy signaling

Recent research showed that NF-κB may represent a pivotal therapeutic approach for sensitizing tumor cells to apoptosis through autophagy [164]. Of note, autophagy is also involved in NF-κB signaling during bone remodeling [165, 166]. The NF-κB pathway can directly augment autophagy by promoting the expression of key autophagy-related proteins, including ATG5, LC3, and Beclin1 [167]. These proteins were reported to be highly involved in osteoclast activity [168, 169], and inhibiting the NF-κB pathway reduced autophagy in pre-osteoclasts [170]. Conversely, NF-κΒ has the potential to inhibit autophagocytosis by either up-regulating the expression of autophagy repressors, like the Bcl-2 family and mTOR, or suppressing autophagy promoters, such as ROS, BNIP3, JNK1, and p53 [171–173]. In turn, autophagy was found to control the NF-κB pathway, primarily by degrading IKK and NF-κB-inducing kinase components [174].

Notably, p62 is implicated in NF-κB signaling and cytoskeletal reorganization. p62 activates NF-κB signaling under stimulation by interleukin 1, RANKL, and nerve growth factor [175], illustrating the pathophysiological significance of NF-κB signaling in autophagy. Recently, therapeutic strategies for bone remodeling have been designed to stimulate the formation of autophagosomes while inhibiting NF-κB signaling and RANKL-induced osteoclast differentiation based on autophagy and NF-κB signaling [165]. In general, the effect of O-GlcNAcylation on bone remodeling through NF-κB signaling depends on changes in autophagy levels.

#### O-GlcNAcylation of ROS/NF-κB/autophagy signaling

O-GlcNAcylation interacts with the NF-κB signaling pathway in various cell types to respond to distinct stress environments [176]. Increased levels of O-GlcNAcylation protein in the heart attenuated the activation of the NF-κB signaling pathway, potentially contributing to ameliorating oxidative stress following hemorrhagic shock [177]. Similarly, increased levels of O-GlcNAcylation exerted a suppressive effect on lipopolysaccharide-induced NF-κB activation in lung tissue [178]. A recent study reported an up-regulation of O-GlcNAcylation during osteoclast differentiation. Additionally, decreasing the O-GlcNAcylation of p65 and nuclear factor of activated T cells c1 (NFATc1) was demonstrated to inhibit their nuclear translocation, consequently hindering osteoclast differentiation [179].

The O-GlcNAcylation of IKKβ at S733 was reported to augment NF-κB activation by preventing phosphorylation [176]. Similarly, elevated O-GlcNAcylation of IKKα and p65 enhanced p65 activity and NF-κB transcription [180], whereas OGT gene knockdown produced the opposite results [181]. Further analysis showed that T322 and T352 were O-GlcNAcylation sites on p65, and the process was facilitated by hyperglycemic circumstances [182]. In addition, NF-κB signaling was inactivated by inhibiting O-GlcNAc production in the inflammatory environment, ultimately increasing the level of autophagy to reduce the inflammatory response [183]. Biomaterials that reduce the activation of NF-κB in BMSCs while enhancing autophagy flux have been developed to treat alveolar bone defects caused by diabetes [184]. Although O-GlcNAcylation is closely related to NF-κB signaling, limited studies have elucidated the mechanism in bone tissue. Thus, these investigations could offer novel insight into bone remodeling under oxidative stress.

## Concluding remarks and perspectives

Long-term exposure to ROS induced by oxidative stress suppresses the survival and proliferation of BMSCs and osteoblasts while contributing to osteoclast differentiation. It is the main process inhibiting bone remodeling in osteoporosis, MRONJ, and bone fractures. During this mechanism, autophagy is activated by oxidative stress signaling pathways and plays a protective role. Existing research demonstrates that O-GlcNAcylation influences autophagy and the expression of antioxidant genes to mitigate oxidative stress, thereby diminishing bone resorption and contributing to bone remodeling. However, the impact of O-GlcNAcylation on autophagy during oxidative stress varies among individuals and depends on the collective influence of diverse disease states and signal transduction pathways.

The precise O-GlcNAcylation modification sites in autophagy-related oxidative stress signaling pathways involved in bone remodeling regulation need further investigation. Identifying these sites may open avenues for designing targeted peptides and nanocarriers, which can be enhanced using lectins or antibodies to actively target O-GlcNAc. Additionally, exploring whether the HBP pathway can regulate O-GlcNAcylation to mitigate oxidative stress-induced osteoporosis in diabetic patients also warrants further investigation. Despite being in its infancy, accumulating evidence suggests that O-GlcNAcylation is a promising therapeutic target and provides novel ideas for diagnostic biomarkers for bone-related disorders.

## Data Availability

No datasets were generated or analysed during the current study.

## Abbreviations

O-GlcNAcylation: O-linked N-acetylglucosamine protein modification
PTM: Post-translational modification
ROS: Reactive oxidative species
BMSCs: Bone marrow-derived mesenchymal stem cells
MRONJ: Medication-related osteonecrosis of the jaw
OGT: O-GlcNAc transferase
OGA: O-GlcNAcase
UDP-GlcNAc: Uridine-5′-diphosphate-N-acetylglucosamine
HBP: Hexosamine biosynthetic pathway
ATG: Autophagy-related
Nrf2: Nuclear factor erythroid 2‐related factor 2
RANKL: Receptor activator of NF-κB ligand ligand
ULK: UNC51-like kinase
FoxO: Forkhead box transcription factor O
VPS15: Vacuolar protein sorting 15
PI3P: Phosphatidylinositol 3-phosphate
LC3: Microtubule-associated protein 1 light chain 3
SNARE: Soluble N-ethylmaleimide-sensitive factor attachment protein receptors
AREs: Antioxidant response elements
Keap1: Kelch‐like ECH‐associated protein 1
CUL3: CULLIN3
HCF1: Host Cell Factor-1
FoxO: Forkhead box class O
TCF: T cell factor
PRG4: Joint lubricant proteoglycan 4
AMPK: AMP­activated protein kinase
Mtor: Mammalian target of rapamycin
4E-BP1: 4E-binding protein 1
S6K1: S6 kinase1
OPG: Osteoprotegerin
NFATc1: Nuclear factor of activated T cells c1
